# Wnt Inhibitors *Dkk1* and *Sost* Are Downstream Targets of BMP Signaling Through the Type IA Receptor (BMPRIA) in Osteoblasts

**DOI:** 10.1359/jbmr.090806

**Published:** 2009-10-05

**Authors:** Nobuhiro Kamiya, Tatsuya Kobayashi, Yoshiyuki Mochida, Paul B Yu, Mitsuo Yamauchi, Henry M Kronenberg, Yuji Mishina

**Affiliations:** 1 School of Dentistry, University of Michigan Ann Arbor, MI, USA; 2 Laboratory of Reproductive and Developmental Toxicology, NIEHS, National Institute of Health Research Triangle Park, NC, USA; 3 Endocrine Unit, Massachusetts General Hospital and Harvard Medical School Boston, MA, USA; 4 Dental Research Center, University of North Carolina at Chapel Hill Chapel Hill, NC, USA; 5 Division of Cardiology, Massachusetts General Hospital and Harvard Medical School Boston, MA, USA; 6 Center for Excellence in Hip Disorders, Texas Scottish Rite Hospital for Children Dallas, TX, USA

**Keywords:** BMP receptor type IA (BMPRIA), Wnt, osteoblasts, Sost, Dkk1

## Abstract

The bone morphogenetic protein (BMP) and Wnt signaling pathways both contribute essential roles in regulating bone mass. However, the molecular interactions between these pathways in osteoblasts are poorly understood. We recently reported that osteoblast-targeted conditional knockout (cKO) of BMP receptor type IA (BMPRIA) resulted in increased bone mass during embryonic development, where diminished expression of *Sost* as a downstream effector of BMPRIA resulted in increased Wnt/β-catenin signaling. Here, we report that *Bmpr1a* cKO mice exhibit increased bone mass during weanling stages, again with evidence of enhanced Wnt/β-catenin signaling as assessed by Wnt reporter *TOPGAL* mice and TOPFLASH luciferase. Consistent with negative regulation of the Wnt pathway by BMPRIA signaling, treatment of osteoblasts with dorsomorphin, an inhibitor of Smad-dependent BMP signaling, enhanced Wnt signaling. In addition to *Sost*, Wnt inhibitor *Dkk1* also was downregulated in cKO bone. Expression levels of *Dkk1*and *Sost* were upregulated by BMP2 treatment and downregulated by Noggin. Moreover, expression of a constitutively active *Bmpr1a* transgene in mice resulted in the upregulation of both *Dkk1* and *Sost* and partially rescued the *Bmpr1a* cKO bone phenotype. These effectors are differentially regulated by mitogen-activated protein kinase (MAPK) p38 because pretreatment of osteoblasts with SB202190 blocked BMP2-induced *Dkk1* expression but not *Sost*. These results demonstrate that BMPRIA in osteoblasts negatively regulates endogenous bone mass and Wnt/β-catenin signaling and that this regulation may be mediated by the activities of *Sost* and *Dkk1*. This study highlights several interactions between BMP and Wnt signaling cascades in osteoblasts that may be amenable to therapeutic intervention for the modification of bone mass density. © 2010 American Society for Bone and Mineral Research

## Introduction

Bone morphogenetic proteins (BMPs), originally discovered as inducers of ectopic bone,(3) are members of the transforming growth factor β (TGF-β) superfamily.(1,2) BMP signals, like those of other TGF-β family members, are mediated by the concerted activation of type I and type II serine-threonine kinase receptors.(4) On engaging BMP ligands in a heteromeric complex, type II receptors activate the kinase function of type I receptors to initiate signaling via intracellular Smads 1, 5, and 8. Of the three type I receptors that recognize BMPs (BMPRIA or ALK3, BMPRIB or ALK6, and ACVRI or ALK2), BMPRIA is the most effective receptor for transducing canonical BMP ligands BMP2(5) and BMP4,(6) which are abundantly expressed in bone.(7)

The osteogenic function of BMPs has been documented extensively, with numerous in vitro studies supporting a critical role of BMP signaling in osteoblastogenesis.(8) While constitutive activation of BMP signaling in muscle appears to induce ectopic ossification in patients with fibrodysplasia ossificans progressiva(9) and in a mouse model,(10) the physiologic effects of BMP signaling on endogenous bone formation in vivo have not been fully elucidated. The early embryonic lethality of conventional knockout mice deficient in BMP ligands and receptors prior to bone development has precluded these studies.(11–13) To circumvent this lethality, we recently generated conditional knockout (cKO) mice in which BMP signaling through BMPRIA was disrupted in a bone-specific and age-dependent manner using a tamoxifen-inducible *Cre/lox* P system under the control of a 3.2 kb type I collagen promoter. In these *Bmpr1a* cKO mice, we unexpectedly observed increased bone mass in embryos, weanlings, and adult animals.(14,15) In cKO adult bones, increased bone mass resulted from severely suppressed bone resorption owing to reduced RANKL-OPG pathway-induced osteoclastogenesis despite a simultaneous small reduction in the rate of bone formation.(15) These findings suggest that BMP signaling in osteoblasts regulates the balance between bone formation and resorption to control bone mass.

Wnt signaling in osteoblasts also plays an important role in regulating bone formation and mass.(16–20) Experiments using pluripotent mesenchymal cell lines to test the interaction between BMP and Wnt signaling in osteoblasts have yielded somewhat contradictory results. BMP2 has been reported to induce both Wnt3a and Wnt/β-catenin signaling,(21–23) whereas Wnt3a, in turn, enhances BMP4 expression.(24) However, Wnt3a also has been reported to repress BMP2-dependent *Id1* expression.(25) In contrast, we recently demonstrated that loss of BMPRIA signaling in osteoblasts downregulates sclerostin/Sost and upregulates Wnt/β-catenin signaling, resulting in increased bone mass during embryonic stages.(14) Our results provide a potential mechanism by which BMP signaling in osteoblasts negatively regulates Wnt signaling to control fetal bone mass.

Since BMPs are used clinically to improve fracture healing,(26) our previous findings of increased bone mass in *Bmpr1a*-deficient mice is unexpected and contrary to the rationale behind the application of BMPs in orthopedics. This study seeks to elucidate further the molecular mechanism by which BMP signaling regulates bone mass. Here we report that loss of BMPRIA in postnatal osteoblasts increases bone mass via upregulation of Wnt/β-catenin signaling. In addition to Sost, we find that Wnt inhibitor Dkk1 is also a downstream target of BMPRIA signaling in osteoblasts, lending further support for a negative regulation of Wnt signaling via BMPRIA.

## Materials and Methods

### Mice and tamoxifen administration

A transgenic mouse line expressing the tamoxifen (TM)–inducible Cre fusion protein Cre-ER™ under the control of a 3.2 kb mouse procollagen *α1(I)* promoter (*Col1-CreER*^*TM*^) was generated by pronuclear injection(14,15) and crossed with floxed *Bmpr1a* mice.(27) TM (T5648, Sigma, St. Louis, MO, USA) was dissolved in a small volume of ethanol, diluted with corn oil at a concentration of 10 mg/mL, and stored at −20°C until use. To generate *Bmpr1a* cKO mice (*Col1-CreER*^*TM*+^*:Bmpr1a*^*fx/fx*^) and littermate controls (*Col1-CreER*^*TM*–^:*Bmpr1a*^*fx/fx*^) in weanling stages, TM (75 mg/kg) was injected intraperitoneally (i.p.) into nursing females every 3 days from P_2_ until euthanasia at P_10_, P_14_, or P_21_.(15) Mice that conditionally express a constitutively active form of *Bmpr1a* (ca*Bmpr1a*), which has a mutation in the glycine and serine residue (GS) regulatory domain of BMPRIA (Q227D) resulting in ligand-independent activation of Smad signaling after Cre recombination, were generated as reported previously(14) and crossed with *Col1-CreER*^*TM*^ mice. After injection of TM into nursing females every 3 days from P_2_ to P_21_, ca*Bmpr1a* mutant mice (*Col1-CreER*^*TM*+^:ca*Bmpr1a*^*+*^) were compared with littermate controls (*Col1-CreER*^*TM*–^:ca*Bmpr1a*^*+*^). Both received TM through milk until sacrifice. *ROSA26* Cre reporter (*R26R*)(28) and *TOPGAL*(29) mice were obtained from Dr. Philippe Soriano and the Jackson Laboratory, respectively. C57BL/6 and CD1 mice were used for isolation of wild-type osteoblasts and calvaria culture. The animal protocol was approved by the Institutional Animal Care and Use Committee (IACUC) at the NIEHS/NIH.

### Histology

For hematoxylin and eosin (H&E) staining, rib cages and skulls were fixed in 4% paraformaldehyde, decalcified with 10% EDTA, and embedded in paraffin. Paraffin sections were cut at 8 µm and stained using a standard protocol. For β-galactosidase (β-gal) staining, decalcified bones were prepared in 30% sucrose before frozen sectioning. Sections were stained with X-gal for β-gal activity and counterstained with eosin. Whole rib bones and skulls also were stained with X-gal as previously.(14,15) For immunofluorescence, osteoblasts from newborn cKO and littermate controls were fixed with 4% paraformaldehyde for 10 minutes, and immunostaining was performed using rabbit polyclonal antibodies against β-gal (1:100, Cappel Laboratories, Inc., Cochranville, PA, USA) and antirabbit IgG labeled with Alexa Fluor 488 (1:1000, Invitrogen, Carlsbad, CA, USA). Nuclei were stained with 4,6-diamidino-2-phenylindole (DAPI) (10 µg/mL, Sigma) for 10 minutes. Then β-gal-positive cells per DAPI-positive cells were calculated (%) in the same field.

### X-ray and DXA analyses

X-ray images of rib cages from P_21_ mice were taken using a Faxitron X-ray system (Faxitron X-Ray, Lincolnshire, IL, USA). Bone mineral density (BMD) was determined by dual-energy X-ray absorptiometry (DXA) using the Lunar PIXImus2 densitometer (GE, Fairfield, CT, USA).

### Quantitative real-time RT-PCR (qRT-PCR)

RNA was isolated from P_21_ rib bones using Trizol (Invitrogen) and from primary osteoblasts using Picopure (Arcturus, Sunnyvale, CA, USA). cDNA was synthesized using the SuperScript Preamplification System (Invitrogen). PCR reactions, data quantification, and analysis were performed according to the manufacturer's standard protocol of TaqMan gene expression assays: *Axin2*: Mm01265783_m1, *Bmpr1a*: Mm00477650_m1, *Ctgf*: Mm01192931_g1, *Dkk1*: Mm00438422_m1, *Lef1*: Mm00550265_m1, *Lrp5*: Mm01227476_m1, and *Sost*: Mm03024247_g1 (Applied Biosystems, Rotkreuz, Switzerland). Values were normalized to *Gapdh* using TaqMan Rodent GAPDH Control Reagents (Applied Biosystems). All measurements were performed in triplicate and analyzed using the 2^−ΔΔ*C*(*t*^ method.(30)

### Primary osteoblast and calvaria culture

Newborn and P_10_ calvariae were digested with type I collagenase (Sigma) and dispase II (Roche, Indianapolis, IN, USA) to isolate osteoblasts, as described previously.(14) Primary osteoblasts were maintained in α-MEM containing 10% fetal bovine serum (FBS) and ascorbic acid (50 µg/mL, Sigma). Primary osteoblasts from wild-type mice were treated with BMP2 for 3 hours at varied concentrations (10, 50, and 100 ng/mL, R&D, Minneapolis, MN, USA). Wild-type osteoblasts also were pretreated with dorsomorphin (10 µM), p38 mitogen-activated protein kinase (MAPK) inhibitor SB202190 (10 µM, Calbiochem, Gibbstown, NJ, USA), and DMSO in the absence of serum for 1 hour before BMP2 treatment (100 ng/mL). For primary osteoblasts from *Bmpr1a* cKO mice or ca*Bmpr1a* mutant mice, 4-hydroxyl tamoxifen (4OH TM, 100 ng/mL, Sigma) was added in culture every other day. For ex vivo bone culture, newborn calvariae from wild-type mice were dissected at the sagittal suture and cultured in modified BGJ (Invitrogen) supplemented with 5% FBS and ascorbic acid (50 µg/mL) for the first 24 hours in culture. Hemicalvariae were treated with 4OH TM (100 ng/mL) and Noggin (100 ng/mL, R&D) in the absence of serum for 5 days.

### Dual luciferase reporter assays

Primary osteoblasts from *Bmpr1a* cKO newborn mice and their littermate controls were plated onto six-well plates at a density of 2 × 10^5^ cells/well containing 10% FBS in α-MEM and grown to 50% to 60% confluence. Cells were transfected with plasmid mixtures containing 2 µg TOPFLASH luciferase construct and 0.05 µg Renilla luciferase driven by the *Drosophila* actin 5C promoter(31) (kindly provided by Dr. Paul A. Wade) using FuGENE 6 Transfection Reagent (Roche) according to the manufacturer's protocol. After 48 hours of transfection, the cells were lysed, and luciferase activity was measured. The values of TOPFLASH luciferase activity were normalized to those of Renilla activity using a dual luciferase assay kit (Promega, Madison, WI, USA). For dorsomorphin treatment, primary osteoblasts from wild-type mice were transfected as described, cultured for 40 hours, treated with DMSO or dorsomorphin (10 µM) and further incubated for 8 hours. The dual luciferase assay was performed in the same manner as described previously. No obvious toxicity was detected in the experiments based on Renilla levels.

### Statistical analysis

All statistical analyses were performed using a two-tailed Student's *t* test.

## Results

### Tissue specificity and efficiency of Cre recombinase in Col1-CreER™ mice

**Fig. 1:**
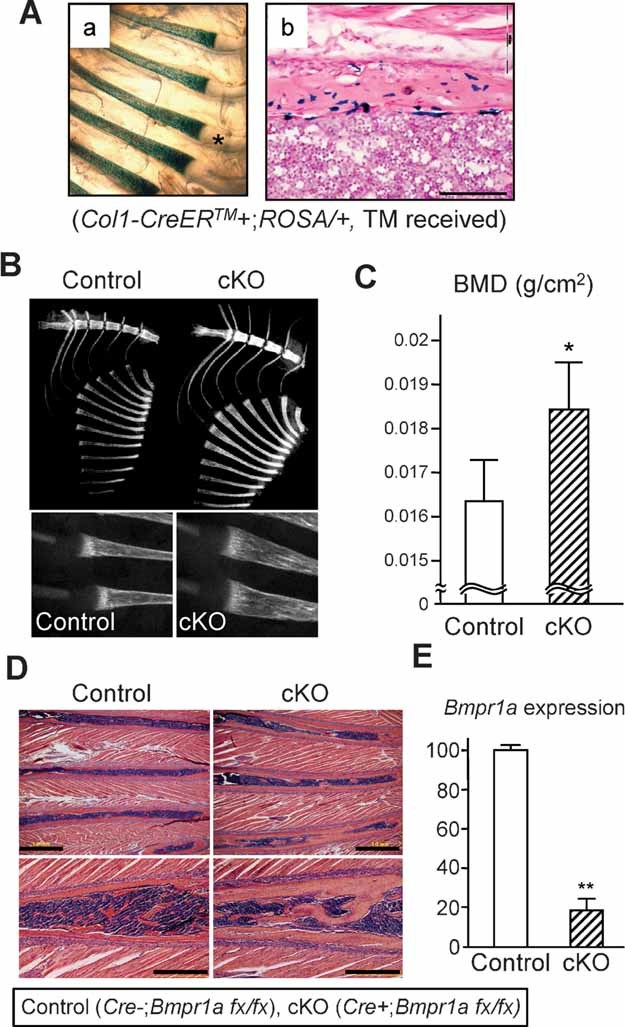
Increased bone mass in *Bmpr1a* cKO weanling mice. Tamoxifen was injected intraperitoneally into nursing females every 3 days from P_2_ to P_20_. (*A*) *ROSA26* Cre reporter mice showed Cre activity on rib bones stained with β-galactosidase (β-gal) at P_21_ (*a*). *Cre*-dependent DNA recombination was detected in osteoblasts in rib bones by β-gal staining (*b*). The asterisk indicates negative staining for β-gal on rib cartilage. Bar: 100 µm. (*B*) Radiodensity of rib bones and sternum was increased in cKO mice (*Cre*^+^, *Bmpr1a^fx/fx^*) compared with controls (*Cre*^–^, *Bmpr1a^fx/fx^*) when assessed by X-ray imaging at P_21_. Rib flaring in cKO mice is shown (*right panel*). (*C*) BMD was determined in ribs using DXA at P_21_. Values are expressed as mean ± SD (control: *n* = 10; cKO: *n* = 12). The asterisk indicates a statistically significant difference between control and cKO (mean ± SD; *t* test; *p < .04). (*D*) H&E staining of P_21_ rib bones. Rib bones were thicker in cKO mice, and bone mass was increased compared with littermate controls in both the diaphysis (*upper panels*) and metaphysis (*lower panels*). Bars: 1 mm (*upper panels*), 500 µm (*lower panels*). (*E*) qRT-PCR analysis for *Bmpr1a* using P_21_ rib bones (mean ± SD; *t* test; **p < .02).

TM-inducible *Col1-CreER*^*TM*^ mice were mated to *ROSA26* reporter mice (*R26R*) to assess Cre activity by staining for β-galactosidase (β-gal) at P_21_. TM was injected intraperitoneally into nursing females every 3 days from P_2_ to P_20_. *Cre* transgenic mice administered TM (*Col1-CreER*^*TM*+^, *ROSA*^*+*^, TM^+^) stained for β-gal in rib bones (Fig. 1A, *a*), where many osteoblasts and osteocytes showed evidence of recombination (see Fig. 1A, b). No staining was observed in cartilage (see Fig. 1A, *a*). At this stage, no recombination was detected in chondrocytes and osteoclasts, as described previously.(15) These results demonstrate that the TM administration delivered through milk specifically and efficiently induces Cre recombination in osteoblasts and osteocytes in weanlings.

### Increased bone mass in BMPRIA cKO rib bones

*Col1-CreER*^*TM*^ mice were bred with mice homozygous for floxed *Bmpr1a* to generate *Bmpr1a* cKO mice (cKO, *Cre*^*+*^*:Bmpr1a*^*fx/fx*^) and wild-type controls (*Cre*^*–*^*:Bmpr1a* ^*fx/fx*^) after maternal TM administration. X-ray analysis demonstrated a modest increase in radiodensity in the rib bones and sternum (see Fig. 1B, *upper panel*) and rib flaring (see Fig. 1B, *lower panels*) in cKO mice at P_21_. BMD measured by DXA was significantly increased in rib bones at P_21_ (see Fig. 1C). Consistent with X-ray imaging and DXA results, H&E staining showed that rib bones in the diaphysis and metaphysis of cKO mice were thicker and had higher bone mass compared with control mice at P_21_ (see Fig. 1D). *Bmpr1a* expression was 80% reduced in cKO rib bones at P_21_, as assessed by qRT-PCR (see Fig. 1E). Bone mass in other bones (i.e., femur, tibia, humerus, tail, and calvaria) also was increased by histology, as previously described,(15) but BMD was unchanged in other bones at this stage (data not shown). Thus we focused on rib bones to investigate the mechanism by which BMP signaling regulates bone mass.

### Upregulation of canonical Wnt signaling in cKO rib bones

**Fig. 2:**
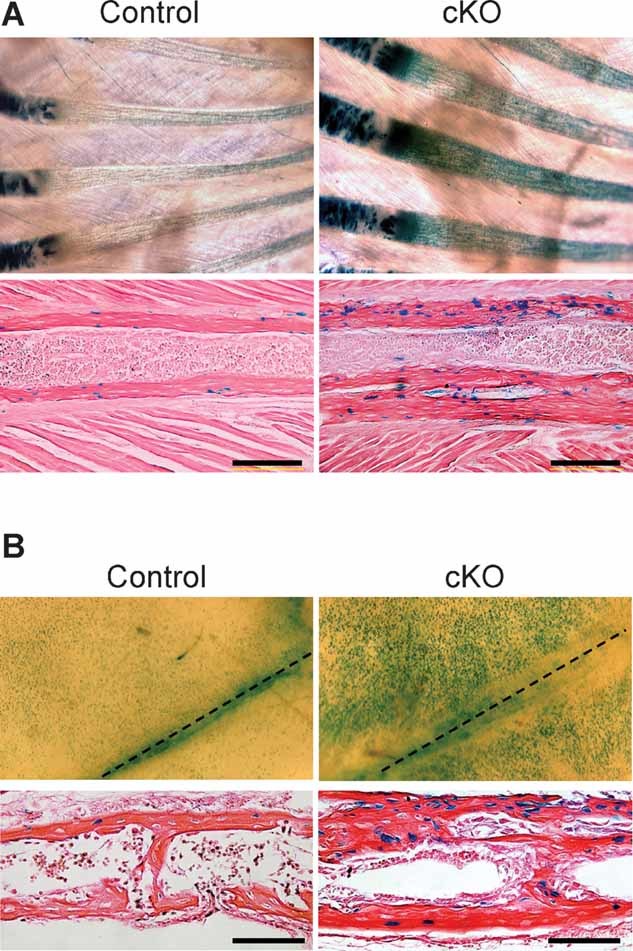
Upregulation of canonical Wnt signaling in *Bmpr1a* cKO mice. (*A*) Canonical Wnt signaling in P_14_ rib bones assessed using *TOPGAL* mice. Whole-mount β-gal staining showed increased staining in cKO rib bones compared with controls (*upper panels*). Histologic analysis showed an increased number of β-gal-positive osteoblasts in cKO rib bones compared with controls (*lower panels*). Bar: 200 µm. (*B*) Canonical Wnt signaling in P_10_ skull bones assessed using *TOPGAL* mice. Whole-mount β-gal staining showed increased staining in cKO calvariae compared with controls (*upper panels*). Histologic analysis showed an increased number of β-gal-positive osteoblasts in cKO calvariae compared with controls (*lower panels*). Dotted lines indicate the sagittal suture. Bar: 100 µm.

Whereas BMP and Wnt signaling are known to independently contribute to bone mass regulation,(17–20) the interaction between these two pathways is incompletely defined and has not been demonstrated previously in vivo. We recently reported that loss of BMPRIA in osteoblasts during embryogenesis upregulates canonical Wnt signaling in mice.(14) To assay canonical Wnt signaling in situ, cKO mice were mated with *TOPGAL* Wnt reporter mice, which express a β-galactosidase transgene driven by a T-cell factor (TCF) β-catenin-responsive promoter.(29) cKO *TOPGAL* mice demonstrated increased Wnt signaling activity in cKO rib bones at P_14_ compared with controls when assessed by whole mount (Fig. 2A, *upper panels*) and sections (see Fig. 2A, *lower panels*) via β-gal staining, as were calvariae at P_10_ and P_14_ (see Fig. 2B; data not shown). Consistent with P_21_ cKO calvariae,(15) P_10_ cKO calvariae also were thicker than controls (see Fig. 2B, *lower panels*). Upregulation of canonical Wnt signaling in cKO rib bones and calvariae thus was found in conjunction with increased bone mass.

**Fig. 3:**
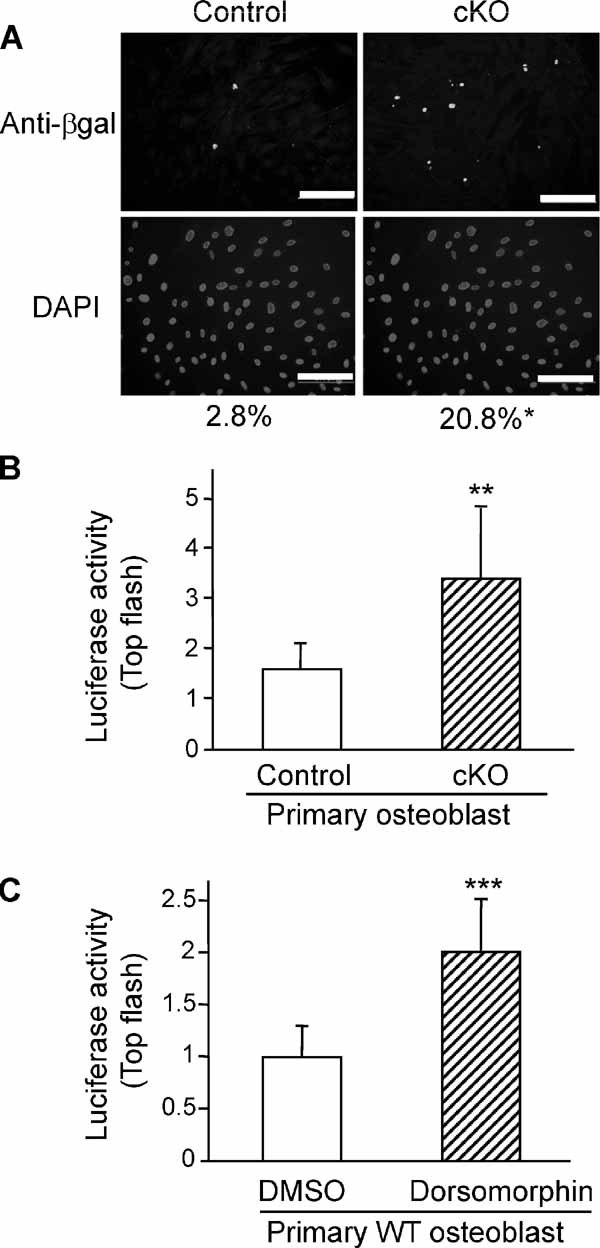
Upregulation of canonical Wnt signaling by loss of BMP signaling in vitro. (*A*) Detection of canonical Wnt signaling in primary osteoblasts from P_10_ *Bmpr1a* cKO and control calvariae (*upper panels*) using *TOPGAL* mice assessed by immunocytochemical staining for β-gal (*green*). Nuclei were stained with DAPI (*blue, lower panels*). Relative ratio of β-gal-positive cell number to DAPI-positive cell number was determined in the same field (mean from 50 fields; *t* test; *p < .01; *n* > 4 per each genotype). Bars: 200 µm. (*B*) TOPFLASH plasmid was cotransfected with pRL-actin vector into primary osteoblasts from newborn *Bmpr1a* cKO and wild-type control calvariae using FuGENE 6 for 48 hours. Transfection efficiency for luciferase activity was normalized to Renilla luciferase (pRL-actin vector) activity. The *y* axis shows relative luciferase units (RLUs, luciferase activity/Renilla units). Promoter activities for *Lef/Tcf*-dependent transcription were increased significantly in cKO osteoblasts (mean ± SD; *t* test; **p < .02). (*C*) Effects of BMP signaling inhibitor dorsomorphin on canonical Wnt signaling in vitro. TOPFLASH plasmid was cotransfected with pRL-actin vector into primary osteoblasts from newborn wild-type calvariae using FuGENE 6. After 40 hours of transfection, these cells were treated with dorsomorphin (10 µM) or DMSO without serum for 8 hours, and then cells were harvested. The *Lef*/*Tcf*-dependent transcriptional activities were increased significantly by the treatment with dorsomorphin, as measured by dual luciferase activity (mean ± SD; *t* test; ***p < .05).

Consistent with findings in cKO rib bones, canonical Wnt signaling activity was enhanced in primary osteoblasts isolated from P_10_ calvariae from cKO mice compared with control osteoblasts (see Fig. 2B). The frequency of β-gal-positive cells was increased significantly in cKO primary osteoblasts compared with control osteoblasts (control: 2.8%, cKO: 20.8%; Fig. 3A). When *Lef/Tcf*-dependent transcriptional activity was assayed using the TOPFLASH reporter system, canonical Wnt signaling was significantly increased in cKO osteoblasts (see Fig. 3B). Corroborating the effects of targeted disruption of BMPRIA in osteoblasts, Wnt signaling activity also was increased significantly (see Fig. 3C) after treating wild-type primary osteoblasts with dorsomorphin, a selective BMP type I receptor inhibitor that effectively blocks BMPRIA-mediated Smad activation under these conditions.(32) Taken together, these findings in primary osteoblasts, calavarial explants, and in situ in transgenic reporter mice confirm that osteoblast BMPRIA signaling negatively regulates canonical Wnt signaling during early postnatal development and that its disruption is associated with increased bone mass.

### Downregulation of *Dkk1* and *Sost* in cKO bones

**Fig. 4:**
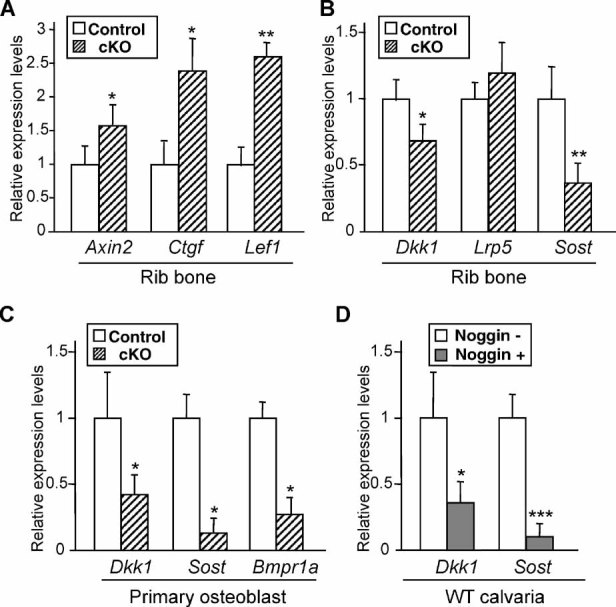
Downregulation of *Dkk1* and *Sost* in *Bmpr1a* cKO rib bones. (*A*) qRT-PCR analysis for Wnt target genes *Axin2*, *Ctgf*, and *Lef1* using P_21_ rib bones. Expression levels of *Axin2*, *Ctgf*, and *Lef1* were increased significantly in cKO rib bones. Values in cKO bones (*striped bar*) are expressed relative to controls (*open bar*). (*B*) qRT-PCR analysis for *Dkk1*, *Lrp5*, and *Sost* using P_21_ rib bones. Expression levels of Wnt inhibitors (*Dkk1* and *Sost*) were reduced significantly in cKO rib bones (*striped bar*), whereas expression of coreceptor *Lrp5* was unchanged. (*C*) qRT-PCR analysis for *Dkk1*, *Sost*, and *Bmpr1a* using primary osteoblasts from P_10_ *Bmpr1a* cKO and control calvariae. Expression levels of *Dkk1*, *Sost*, and *Bmpr1a* were reduced significantly in cKO osteoblasts (*striped bar*) compared with control osteoblasts (*open bar*). (*D*) Suppressed expression of *Dkk1* and *Sost* by Noggin treatment ex vivo, as assessed by qRT-PCR. Newborn calvariae from wild-type mice were treated with Noggin (100 ng/mL) for 5 days. The asterisk indicates a statistically significant difference between Noggin treatment (Noggin^+^) and nontreatment (Noggin^–^) from three independent experiments. (*A–D*) Mean ± SD; *t* test; *p < .05; **p < .02; ***p < .01.

To confirm the findings of enhanced canonical Wnt signaling in cKO tissues and primary cultures, expression of Wnt-related target genes was examined by qRT-PCR using rib bones and primary osteoblasts isolated from calvariae. In P_21_ cKO rib bones, expression levels of Wnt target genes *Axin2*, *Ctgf*, and *Lef1* were increased significantly (Fig. 4A). It has been reported that osteoblasts secrete several proteins into bone extracellular matrix (ECM) that inhibit Wnt signaling and that these ECM molecules can regulate bone mass along with coreceptors.(33–38) We reported that sclerostin/Sost, a Wnt inhibitor, plays an important role in regulating bone mass as a downstream target molecule of BMP signaling during embryonic bone development.(14) In P_21_ cKO rib bones, expression levels of *Sost* and *Dkk1* mRNAs were reduced significantly, whereas levels of Wnt ligands (*Wnt3a*, *Wnt5a*, *Wnt7a*, *Wnt7b*, and *Wnt9a*), other Wnt inhibitors including *Dkk2*, secreted frizzled-related proteins (*sFrp*), and coreceptor *Lrp5* were unchanged (see Fig. 4B; data not shown). In cKO primary osteoblasts, where *Bmpr1a* expression was reduced significantly, *Sost* and *Dkk1* mRNA expression levels also were reduced compared with controls (see Fig. 4C), whereas Wnt target genes were increased (data not shown). Consistent with a positive regulatory effect of BMP signaling on Wnt antagonists, treatment of wild-type calvariae with Noggin, a BMP2 and BMP4 antagonist, inhibited both *Sost* and *Dkk1* expression ex vivo (see Fig. 4D), upregulated Wnt signaling,(14) and increased the expression of Wnt target genes (data not shown). These results obtained from two different types of bone (i.e., rib bone and calvaria) and primary cultures suggest that loss of BMPRIA signaling enhances canonical Wnt signaling during postnatal bone development in osteoblasts via decreased expression of *Dkk1* and *Sost*.

### Positive regulation of *Dkk1* and *Sost* by BMP2

**Fig. 5:**
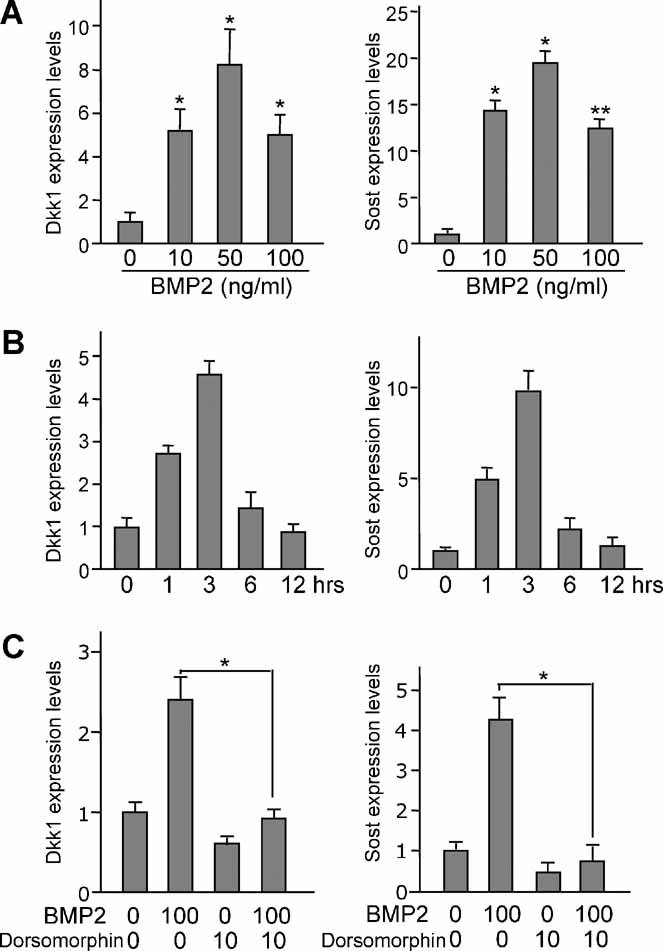
Positive regulation of *Dkk1* and *Sost* expression by BMP2. Primary osteoblasts were isolated from wild-type newborn calvariae. (*A*) Dose-dependent effects of BMP2 on *Dkk1* and *Sost* expression as assessed by qRT-PCR. mRNA was isolated from wild-type osteoblasts treated with BMP2 at the indicated concentration for 3 hours. Values are expressed relative to nontreated osteoblasts (mean ± SD; *t* test; *p < .05; **p < .01). (*B*) Time course of *Dkk1* and *Sost* expression levels using wild-type osteoblasts treated with BMP2 (100 ng/mL). (*C*) Effects of pretreatments of primary osteoblasts with Smad-dependent inhibitor dorsomorphin. mRNA was isolated from wild-type osteoblasts pretreated with dorsomorphin (10 µM) or DMSO in the absence of serum for 1 hour prior to the addition of BMP2 (100 ng/mL) or PBS for 3 hours. Upregulation of *Dkk1* and *Sost* expression by BMP2 treatment was restored by dorsomorphin pretreatment, as assessed by qRT-PCR. Values are expressed relative to osteoblasts without dorsomorphin and BMP2 (mean ± SD; *t* test; *p < .05).

We next investigated a potential link between expression of Wnt inhibitors *Sost* and *Dkk1* and BMP signaling using wild-type primary osteoblasts. In primary osteoblasts treated with canonical BMPRIA ligand BMP2,(5) levels of Dkk1 and Sost increased up to 8- and 20-fold, respectively, after 3 hours, as assessed by qRT-PCR (Fig. 5A). Peak expression levels of Dkk1 and Sost were detected at 3 hours after BMP2 treatment (100 ng/mL) (see Fig. 5B). Upregulation of *Dkk1* and *Sost* expression by BMP2 treatment (100 ng/mL) was blocked by pretreatment with dorsomorphin (see Fig. 5C), which selectively inhibits Smad activation without inhibiting BMP-induced activation of MAPK p38.(32) These results suggest that *Dkk1* and *Sost* expression are regulated by BMP signaling, at least in part via Smad-dependent signaling.

### Upregulation of *Dkk1* and *Sost* by Smad-dependent BMPRIA signaling

**Fig. 6:**
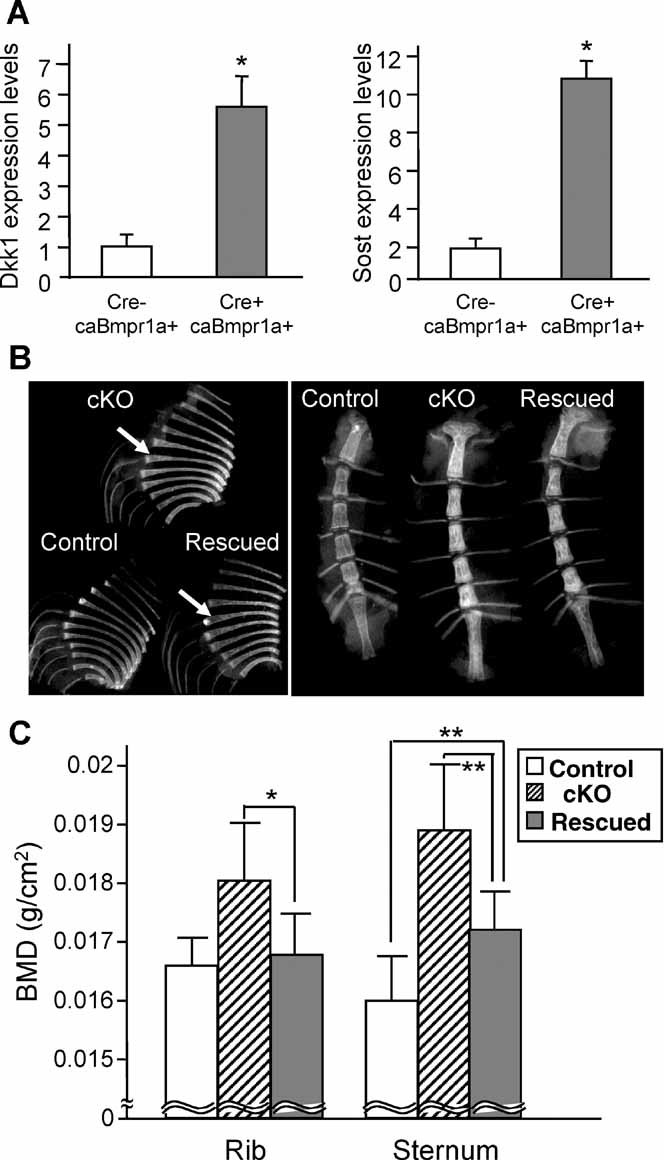
Effects of Smad-dependent *Bmpr1a* signaling on *Dkk1* and *Sost* expression. (*A*) Positive regulation of *Dkk1* and *Sost* expression by overexpressing Smad-dependent *Bmpr1a* signaling. Primary osteoblasts were isolated from constitutively active *Bmpr1a* (ca*Bmpr1a*) newborn mice or littermate controls (*Cre*–, ca*Bmpr1a*^+^*)* and were treated with 4-hydroxyl tamoxifen (100 ng/mL). Both *Dkk1* expression and *Sost* expression were increased significantly more than 5- and 10-fold, respectively, in the ca*Bmpr1a* mutant osteoblasts (*Cre*^+^, ca*Bmpr1a*^+^*)* compared with controls (*Cre*^–^, ca*Bmpr1a*^+^*)*, as assessed by qRT-PCR. Values of the mutant osteoblasts (*striped bar*) are expressed relative to controls (*open bar*) (mean ± SD; *t* test; *p < 0.01). (*B*) Radiodensity of rib bones and sternum from rescued mice expressing ca*Bmpr1a* on a *Bmpr1a* cKO background (*Cre*^+^, ca*Bmpr1a*^+^, *Bmpr1a^fx/fx^*), littermate *Bmpr1a* cKO mice (*Cre*^+^, ca*Bmpr1a*^–^, *Bmpr1a^fx/fx^*) and littermate controls (*Cre*^–^, ca*Bmpr1a*^+^, or *Bmpr1a^fx/fx^*) at P_21_ when assessed by X-ray imaging. White arrows indicate rib flaring observed in cKO and rescued mice. (*C*) BMD as determined by DXA using ribs from control (*open bar*), cKO (*striped bar*), and rescued mice (*gray bar*) at P_21_. BMD of rescued sternums was reduced significantly by 10% compared with cKO sternum and increased by 6% compared with controls. Values are expressed as mean ± SD (*n* > 6; *t* test, *p = .07, **p < .05).

On ligand binding, BMPRIA phosphorylates BMP-responsive Smads (Smad1, Smad5, and Smad8) to induce nuclear translocation.(39) The introduction of certain mutations in the GS regulatory domain of BMPRIA is thought to render the receptor protein active independent of ligand, resulting in “constitutively active” *Bmpr1a* (ca*Bmpr1a*).(40) To confirm whether our observations on the effects of Smad-dependent BMPRIA signaling on *Dkk1* and *Sost* expression were recapitulated in vivo, we generated inducible transgenic mice that conditionally express ca*Bmpr1a*, as we described previously.(14) These mice were crossed with *Col1-CreER*^*TM*^ mice, primary osteoblasts were obtained from newborn calvariae, and then Smad-dependent BMPRIA signaling was induced by administration of 4OH TM into the culture medium. Both *Dkk1* expression and *Sost* expression assessed by qRT-PCR were significantly increased, 5- and 10-fold, respectively, in ca*Bmpr1a* osteoblasts (*Cre*^+^, ca*Bmpr1a*^+^*)* compared with controls (*Cre*^–^, ca*Bmpr1a*^+^*)* (Fig. 6A). We next generated compound transgenic-knockout mice expressing ca*Bmpr1a* on a *Bmpr1a* cKO background (rescued: *Cre*^+^, ca*Bmpr1a*^+^, *Bmpr1a*^*fx/fx*^) and compared them to littermate *Bmpr1a* cKO mice (cKO: *Cre*^+^, ca*Bmpr1a*^–^, *Bmpr1a*^*fx/fx*^) or controls (*Cre*^–^, ca*Bmpr1a*^+^, *Bmpr1a*^*fx/fx*^). X-ray imaging demonstrated a moderate reduction in radiodensity of the rib bones and sternum obtained from compound transgenic-knockout mice compared with *Bmpr1a* cKO mice at P_21_ (see Fig. 6B). In addition, the observation of increased BMD in cKO ribs and sternum was abrogated by enhanced Smad-dependent BMPRIA signaling at P_21_ (see Fig. 6C). These results using inducible compound ca*Bmpr1a* transgenic and *Bmpr1a* cKO mice demonstrate that both *Dkk1* expression and *Sost* expression are positively regulated by BMPRIA-mediated Smad signaling in vivo, with effects on bone mineral density that are most concordant with effects on Wnt pathway regulation.

### Effects of activation of a non-Smad pathway on *Dkk1* and *Sost* expression

**Fig. 7:**
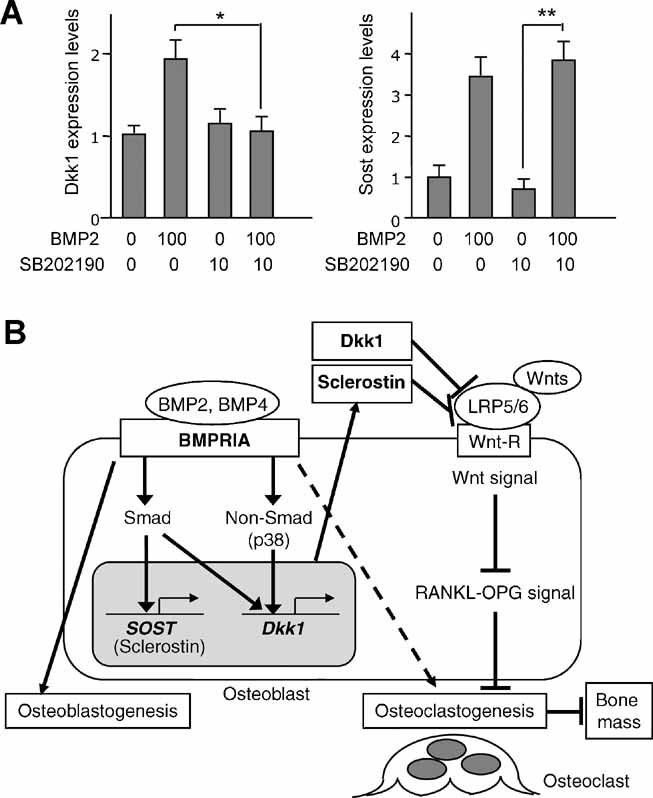
Effects of non-Smad signaling on *Dkk1* and *Sost* expression in osteoblasts. (*A*) Effects of p38 MAPK inhibitor SB202190 on *Dkk1* and *Sost* expression using primary osteoblasts from newborn wild-type mice as assessed by qRT-PCR. mRNA was isolated from wild-type osteoblasts pretreated with SB202190 (10 µM) or DMSO in the absence of serum for 1 hour prior to the addition of BMP2 (100 ng/mL) or PBS for 3 hours. Upregulation of *Dkk1* expression by BMP2 treatment was restored by SB202190 pretreatment. *Sost* expression was increased significantly by BMP2 treatment with SB202190 pretreatment. Values are expressed relative to untreated osteoblasts (neither SB202190 nor BMP2) (mean ± SD; *t* test; *p < .05; **p < .01). (*B*) A proposed model of the relationship between BMPRIA and canonical Wnt signaling through Dkk1 and sclerostin/Sost in bone. BMPRIA signaling upregulates *Sost* expression primarily through Smad-dependent signaling while upregulating *Dkk1* expression through both Smad and non-Smad signaling (p38 MAPK). Both Dkk1 and sclerostin/Sost inhibit canonical Wnt signaling, leading to a decrease in bone mass. Dkk1 and sclerostin/Sost play an important role in regulating bone mass as downstream effectors of BMPRIA signaling.

Rib flaring was still observed in the compound transgenic knockout mice (see Fig. 6B), and the BMD of the rescued sternum was significantly higher than that of control sternum (see Fig. 6C). The partial rescue of the cKO phenotype by the constitutively active transgene led us to postulate that loss of BMP signaling through Smad-independent pathways in addition to the Smad-dependent pathway may contribute to the cKO bone phenotype. BMP signaling can activate intracellular effectors, in particular p38 MAPK, via Smad-dependent pathways both in vitro and in vivo.(41–45) To investigate whether regulation of *Dkk1* and *Sost* expression by BMPRIA signaling requires activation of p38 MAPK, we pretreated primary wild-type osteoblasts with a p38 MAPK inhibitor SB202190 (10 µM) before BMP2 treatment (100 ng/mL). Upregulation of *Dkk1* expression by BMP2 treatment was blocked by SB202190 pretreatment, as assessed by qRT-PCR (Fig. 7A). In contrast, *Sost* expression was increased significantly by BMP2 treatment even after SB202190 pretreatment (see Fig. 7A). These results suggest that *Dkk1* expression is regulated by both Smad-mediated and p38 MAPK signaling, whereas *Sost* expression is regulated mainly by Smad signaling (see Fig. 7B).

## Discussion

We previously reported that loss of BMPRIA-mediated signaling resulted in increased bone mass in embryos and adults(14,15) and discovered that sclerostin*/*Sost is a downstream target of BMPRIA signaling during embryogenesis that inhibits Wnt to regulate bone mass.(14) In this study we found that Dkk1, another Wnt inhibitor, is also a downstream target of BMPRIA signaling at the weanling stage. Based on our findings, we propose a mechanism by which Dkk1 is regulated by signaling through both Smad-dependent and Smad-independent pathways in osteoblasts during postnatal development, whereas Sost is regulated predominantly by the Smad pathway (see Fig. 7).

There is a growing body of evidence to suggest that the BMP and Wnt signaling pathways regulate one another synergistically or antagonistically in a context and age-dependent manner.(46–49) The interplay of Wnt/β-catenin and BMP signaling pathways has not been demonstrated previously during bone development in vivo. However, we recently reported that BMP signaling in osteoblasts downregulates canonical Wnt signaling during embryonic bone development.(14) Consistent with the effects of BMP signaling during embryogenesis, we presently found evidence for negative regulation of canonical Wnt signaling by BMPRIA signaling in osteoblasts postnatally (see Fig. 2). Our findings that embryonic and postnatal BMPRIA signaling negatively regulate canonical Wnt signaling in osteoblasts agree with other studies that have shown an inhibitory effect of BMP signaling on Wnt signaling in lungs,(50) intestines,(49) hair,(51) and joints.(48) Although many mouse models and human mutations modifying the Wnt signaling pathway have been described, no studies to date have addressed the in vivo interaction of BMP and Wnt signaling in osteoblasts.

Recent studies using pluripotent mesenchymal cell lines have suggested that BMP signaling may upregulate Wnt signaling to synergistically regulate osteoblastogenesis,(23,52) possibly through an autocrine or paracrine loop.(21) This is in contrast with our mouse studies showing that BMP signaling downregulates Wnt signaling. The discrepancy may be due to the use of mesenchymal cell lines rather than primary osteoblasts or the supraphysiologic levels of in vitro BMP treatment or reflect the coordinated action of multiple cell types including osteoclasts in a physiologic setting compared with cell monoculture. The potential importance of the physiologic context of these pathways may be reflected in the finding that canonical Wnt signaling was markedly upregulated in cKO mice (see Fig. 2B, C) but only modestly increased in isolated *Bmpr1a*-deficient osteoblasts (see Fig. 3A, B).

In mice, both *Sost* and *Dkk1* expression levels were downregulated by disrupting *Bmpr1a* (see Fig. 4) and upregulated by enhancing BMP signaling via ca*Bmpr1a* (see Fig. 6). These results, which were replicated in cell culture (see Fig. 5A), suggest that both Dkk1 and sclerostin/Sost are downstream targets of BMPRIA. Previous reports support this notion because BMPRIA ligands BMP2 and BMP4 induce *Dkk1* expression during limb development in mice and chickens,(53,54) as well as *Sost* expression in mouse and human osteoblasts.(55,56) In parallel, inhibition of BMP signaling with dorsomorphin suppressed the expression of *Dkk1* and *Sost* (see Fig. 5B), resulting in upregulated canonical Wnt signaling (see Fig. 3C). Noggin also inhibited *Dkk1* and *Sost* expression (see Fig. 4D), consistent with previous reports that Noggin inhibited *Sost* expression with upregulation of Wnt signaling in mice(14) and suppressed *Dkk1* expression in chickens.(54) Although *Dkk1* has been proposed to be a target of BMP signaling in mouse limb development,(53) this study provides the first evidence to our knowledge that BMPRIA regulates *Dkk1* expression in osteoblasts. The finding that both Smad-dependent and Smad-independent pathways appear to contribute to *Dkk1* expression, whereas *Sost* requires only Smad-dependent signaling suggests differential regulation of these genes by BMPRIA.

Both Dkk1 and sclerostin/Sost are expressed by osteoblasts as secreted proteins that inhibit Wnt/β-catenin signaling by binding to coreceptors low-density lipoprotein receptor–related protein 5 and 6 (LRP5 and LRP6).(36,37,57) Conventional knockouts of *Dkk1* die in utero from defective head induction and limb formation.(53) Mice heterozygous for *Dkk1* (*Dkk1*^+/−^ mice) exhibit a high-bone-mass (HBM) phenotype,(58) whereas overexpression of *Dkk1* in osteoblasts causes osteopenia.(59) In addition, increased *DKK1* expression in bone marrow also has been associated with lytic bone lesions in patients with multiple myeloma.(60) Collectively, these results support the hypothesis that Dkk1 functions as a potent negative regulator of bone mass. *Sost* KO mice are viable and exhibit increased bone mass,(61) similar to *Dkk1*^+/−^ mice. In humans, loss-of-function and hypomorphic mutations in *SOST* cause sclerosteosis(62,63) and Van Buchem disease,(64,65) respectively, with an HBM phenotype. Consistent with these observations, *Bmpr1a* cKO mice, which are deficient in *Dkk1* and *Sost* expression, show an HBM phenotype. Furthermore, increased expression of *Dkk1* and *Sost* in osteoblasts by constitutively activated BMPRIA signaling is associated with partial rescue of the *Bmpr1a* cKO bone phenotype. Our present data support the interpretation that Dkk1 and sclerostin/Sost act physiologically as inhibitors of canonical Wnt signaling and therefore as negative regulators of bone mass in mice.

Both Smad-dependent and Smad-independent pathways are important for BMP signaling. p38 MAPK plays a central role in Smad-independent pathways of TGF-β/BMP signaling(41–45) by accelerating osteoblastogenesis,(66,67) which can be repressed by p38 MAPK inhibitors.(68,69) This study demonstrated that the p38 MAPK inhibitor SB202190 prevented the upregulation of *Dkk1* by BMP2 but did not alter *Sost* expression in osteoblasts (see Fig. 7A). This is consistent with a previous report showing that upregulation of *Dkk* by BMP5 was severely impaired after cotreatment with p38 MAPK inhibitors in chick limb buds.(42) The p38 MAPK pathway thus appears to contribute at least part of the regulation of Wnt signaling by BMPRIA. Given the demonstrated importance of p38 MAPK signaling in osteoblastogenesis, it is highly plausible that additional Smad-independent mechanisms may link BMP signaling and Wnt signaling in this lineage.

The Food and Drug Administration (FDA) has approved BMP2 and BMP7 for clinical use in long bone open fractures, nonunion fractures, and spinal fusion. However, despite significant evidence of their potential for bone regeneration in animal and preclinical studies, the current clinical data supporting the effectiveness of BMP therapy is somewhat unconvincing.(70,71) The lack of a clear benefit from BMPs might stem from an incomplete understanding of the variable and context-sensitive effects BMPs exert on diverse cell types in bone, including chondrocytes, osteoblasts, and osteoclasts. Studies focusing on BMP receptors in chondrocytes including mesenchymal cells suggest that these cells respond to BMP signaling by increasing bone mass during the endochondral formation process.(72–75) In contrast, when we examined function of BMPRIA in osteoblasts with respect to bone mass determination, BMP signals consistently inhibited canonical Wnt signaling and bone mass while exerting concordant effects on *Dkk1* and *Sost*. Our current evidence adds an important nuance to the conventional notion of BMPs as inducers of osteogenesis and positive regulators of bone mass owing to their inverse effects on Wnt signaling in osteoblasts. The functions of BMPRIA signaling in osteoclasts remain largely unknown and merit future study, although we previously observed a significant decrease in osteoblast-dependent osteoclastogenesis in cKO mice.(14,15) This revision of our understanding of the BMP signaling pathway in clinical therapeutics might suggest that in some circumstances BMP inhibition would be desirable for promoting bone mass. Understanding the complex roles of the BMP signaling pathway in chondrocytes, osteoblasts, osteoclasts, and other cell types that contribute to bone development, homeostasis, and remodeling not only will help to improve our knowledge of the dynamic processes that are perturbed in settings of bone fracture, ovariectomy, orchiectomy, mechanical loading, and aging but also may provide novel therapeutically useful strategies.
